# Transformative Innovations in Reproductive, Maternal, Newborn, and Child Health over the Next 20 Years

**DOI:** 10.1371/journal.pmed.1001969

**Published:** 2016-03-02

**Authors:** Cyril M. Engmann, Sadaf Khan, Cheryl A. Moyer, Patricia S. Coffey, Zulfiqar A. Bhutta

**Affiliations:** 1 Maternal, Newborn, Child Health and Nutrition, PATH, Seattle, Washington, United States of America; 2 Department of Pediatrics, Neonatology, University of Washington, Seattle, Washington, United States of America; 3 Departments of Learning Health Sciences and Obstetrics & Gynecology, University of Michigan Medical School, Ann Arbor, Michigan, United States of America; 4 Device/Tools Global Program, PATH, Seattle, Washington, United States of America; 5 Center for Global Child Health, Sick Kids, Toronto, Ontario, Canada; 6 Center of Excellence in Women and Child Health, Aga Khan University, Karachi, Pakistan

## Abstract

As part of the "Grand Convergence: Aligning Technologies and Realities in Global Health" Collection, Cyril Engmann and colleagues discuss promising innovations that have the potential to move the RMNCH agenda forward.

**Summary Points**

- Accelerating progress in reproductive, maternal, newborn, and child health (RMNCH) over the past 30 years has resulted in significant decreases in mortality, as well as shifts in causes of death. For example, deaths from diarrhea among children under age 5 have significantly declined. This increased survival means an increasing fraction of under-5 deaths occur in the first 4 weeks of life, the neonatal period.
- Transformative changes, including advances such as the development of immunizations, wide uptake of contraception, and the availability of medications such as oxytocin, have contributed to an improved morbidity and mortality curve. Such advances are set against a broader backdrop of increasing national wealth, stronger health systems, aligned political agendas, and advocacy systems.
- Global mechanisms and strategies such as the Global Strategy for Women’s, Children’s, and Adolescents’ Health, Global Alliance for the Vaccine Initiative (GAVI), the United Nations Commission on Life-Saving Commodities for Women and Children, Family Planning 2020, and the Every Newborn Action Plan, among others, are serving to drive the global agenda forward, although stubborn gaps remain.
- In this paper, we discuss promising innovations that in our opinion have significant promise in moving the RMNCH agenda forward. While some of these are technologies, others are efforts aimed at improving commodities, increasing demand for services, and promoting equity in access.

## Introduction

Reproductive, maternal, newborn, and child health (RMNCH) was a pivotal focus of the Millennium Development Goals (MDGs). On the cusp of the Sustainable Development Goals’ (SDGs’) era to guide progress for the next 20 years, RMNCH continues to be central to the SDG targets that have been set. Building on the *Lancet* “Commission on Investing in Health” publication, we reflect on major levers that have resulted in increased RMNCH survival over the past 25–30 years [1] and examine promising and important innovations in RMNCH that have transformative potential for the survival and well-being of mothers and children worldwide.

### RMNCH Yesterday and Today

The epidemiology of reproductive, maternal, newborn, and child mortality has changed significantly over the past 25 years [2,3]. In high-income settings, maternal mortality has more than halved, while the decline has been less in low- and middle-income countries. Especially in sub-Saharan Africa, maternal mortality rates plateaued and even increased during the HIV-AIDS epidemic [4,5]. In child health, global under-5 deaths nearly halved from 12.2 million in 1990 to 6.3 million in 2013 [3]. Closer inspection suggests that nine countries (India, China, Pakistan, Bangladesh, Indonesia, Afghanistan, Brazil, Nigeria, and Ethiopia) were responsible for two-thirds of these declines. While overall global trends show a steady decline, the causes and distribution of deaths have changed significantly over time [6]. Diarrhea and pneumonia, once leading causes of under-5 mortality, continue to decrease at remarkable rates in certain settings, and neonatal mortality now accounts for more than 44% of all under-5 deaths [7]. Among neonatal deaths, prematurity is the most common cause of mortality [8].

The landscape of global RMNCH today is very different from what it was 30 years ago. Thirty years ago, the MDGs were not articulated, and neither the Global Fund nor the Global Alliance for Vaccine and Immunization (GAVI) existed. “Mega-billanthropy,” through vehicles such as the Giving Pledge, in which billionaires commit to giving away half of their wealth during their lifetime, had yet to be conceptualized. Official development assistance (ODA) for health stood at US$6.7 billion in 1990, compared to US$28.4 billion in 2011 [9]. Within maternal, newborn, and child health (MNCH) alone, the total volume of worldwide ODA more than doubled between 2003 and 2010, rising from US$2.6 billion in 2003 to US$6.5 billion in 2010 [10].

**Fig 1:**
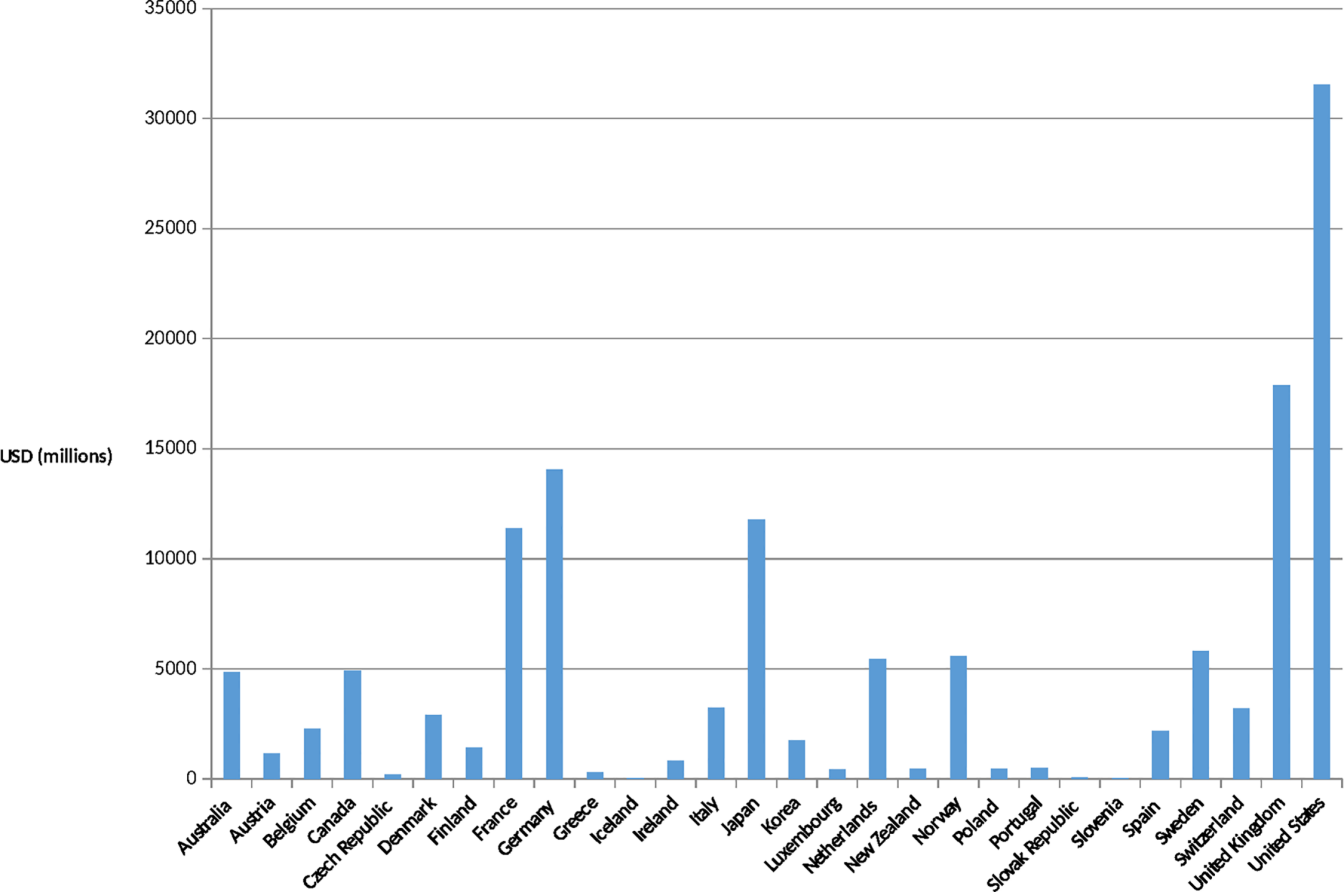
Development Assistance Committee (DAC) members' official development assistance (ODA) in 2013. Source: OECD. Development Co-operation Report 2014: Mobilising Resources for Sustainable Development. Paris: OECD Publishing; 2014. Available at http://observ-ocd.org/sites/observ-ocd.org/files/publicacion/docs/informe_coop.desen_._2014_ocde.pdf. Accessed January 14, 2016.

**Fig 2:**
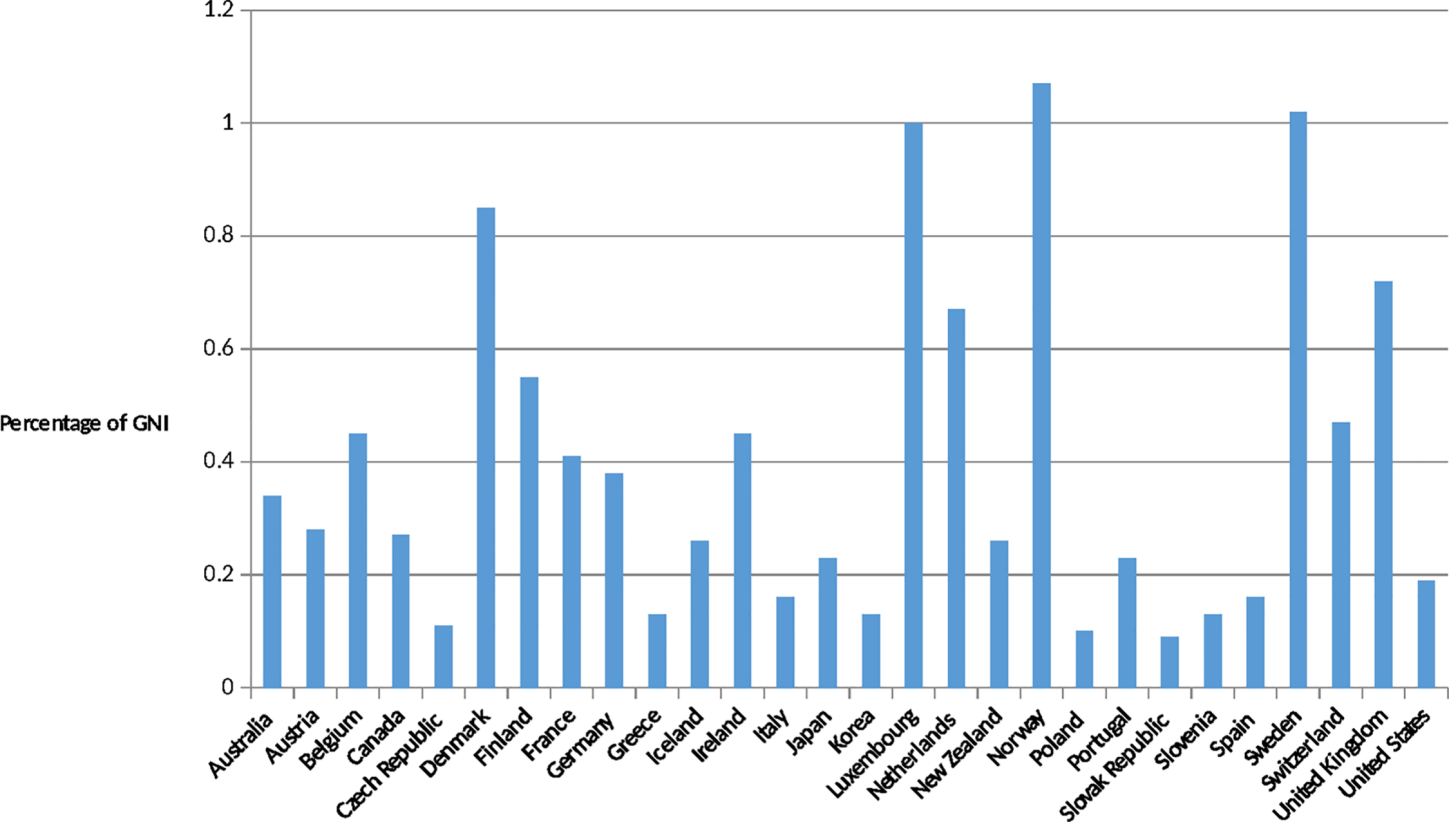
ODA as a percentage of gross national income (GNI) in (2013). Source: OECD. Development Co-operation Report 2014: Mobilising Resources for Sustainable Development. Paris: OECD Publishing; 2014. Available at http://observ-ocd.org/sites/observ-ocd.org/files/publicacion/docs/informe_coop.desen_._2014_ocde.pdf. Accessed January 14, 2016.

Country-level improvements have also been significant over the past 30 years. Many countries have undergone remarkable economic improvements, which in many cases led to more robust health systems. Leaders of African Union countries signed the Abuja Declaration in 2001, pledging to spend 15% of their annual budgets on health by 2015. While only a minority of African countries appear to have realized that pledge as of 2015, there are powerful examples of success: Rwanda spends nearly 22% of its national budget on health [11]. In India, net ODA received as a percentage of gross national income was 0.1% in 2013, compared to 2.1% in 1964, a 20-fold difference [12]. In March 2015, the British Parliament became only the sixth of the 29 wealthy nations who are members of the Development Assistance Committee (DAC) to enact the promise made in 1970 to spend 0.7% of its gross national products on international aid, and the United Arab Emirates now gives 1.25% of its gross national product, a 4-fold increase since the previous year [13]. The United States contribution to ODA is now estimated at US$31 billion, a significant increase over previous years, although short of the UN target of 0.7% (Figs 1 and 2).

However, many successes in RMNCH cannot be attributed to condition-specific interventions alone, i.e., interventions solely and specifically directed at one disease or condition. Among many other factors, there have been accompanying improvements in country revenues, health financing, education and food security systems, and alignment of global and national political and advocacy purposes, as well as an absence of war/strife, that together have successfully accelerated progress or “bent the curve” in reducing reproductive maternal, newborn, and child mortality. Some examples are instructive to consider, particularly because until their development, a huge toll in mortality and morbidity was exacted.

#### Increased access to and uptake of contraception and family planning services and prevention of unsafe abortion practices

Major reductions in maternal and newborn morbidity and mortality have been noted with uptake of family planning services and increased birth spacing. Estimations are that fulfilling the unmet need for modern family planning methods will avert 70,000 maternal deaths (18,000 attributable to unsafe abortion and 53,000 from complications of pregnancy and childbirth) and 500,000 newborn deaths every year [14]. In addition, improvements in training, care provision, and methods have reduced mortality due to unsafe abortion from 69,000 in 1990 to 47,000 in 2008 [15]. This trend is likely to continue, given the increasingly wide availability of drugs to induce a pharmacologic abortion, diminished need for surgical interventions post delivery and post abortion, and the increasing thrust of the global community to make family planning options available to millions of women worldwide.

#### Increasing immunization coverage

The introduction of immunizations over the past century has been one of the biggest successes of public health and a major lever in reducing under-5 mortality. Vaccination with diphtheria-tetanus-pertussis (DPT), polio, measles, and Bacillus Calmette–Guérin (BCG) currently saves an estimated 3 million lives each year [16,17], smallpox has been eradicated, and the world stands on the brink of eradicating polio. Certain galvanizing programs such as the expanded program on immunizations (EPI) and organizations such as GAVI and the Global Fund have further spurred on these wins [18]. The success of maternal tetanus administration in reducing the incidence of neonatal tetanus (and more recent data on the benefits of maternal immunization with influenza vaccine on newborn mortality and morbidity) has initiated a promising line of inquiry into broader applications of maternal immunization [19].

#### Improvements in obstetric care and improved understanding and management of intrapartum birth asphyxia among newborns

Over the past 40 years, ultrasound and doppler use have revolutionized obstetric practice and produced a clearer understanding of the pathophysiology of intrapartum birth asphyxia. An increasing number of standardized neonatal resuscitation protocols assist in promptly identifying and addressing the nonbreathing baby [20]. In many countries, these protocols have been applied by skilled birth attendants (and in some cases by nonskilled birth attendants), whose presence early in the process can dramatically improve neonatal outcomes [21].

Though we have highlighted these interventions for their health impact in RMNCH, many of these interventions gained traction against a backdrop of significant social and economic changes, such as increasing labor force participation of women and the need for birth spacing and limiting.

### Looking Forward

While these interventions have changed the RMNCH landscape over the past 25 years, what might be “game-changing innovations” over the next 25 years to not only increase survival but to also improve healthy development, well-being, and thriving, the combination described programmatically by some as “thrival”? We posit innovations within the following categories: technology, commodities, demand-side barriers to care seeking and care provision, supply-side issues (especially for marginalized populations), financing, and monitoring and evaluation to ensure accountability and real-time feedback for quality assurance and continuous quality improvement. We recognize that the success of the technological and nontechnological interventions discussed in the ensuing sections is and will often be dependent on nonbiomedical factors. For the purposes of this paper and its scope, we will restrict the discussion to a biomedical focus.

## Technological

By 2035, the technological advances in health care provision are likely to be myriad. Within RMNCH, a few key innovations already in the development pipeline today could dramatically increase survival and well-being. In low-income countries, two conditions that affect both the mother and her baby during pregnancy and result in maternal and perinatal mortality and morbidity are infections and preeclampsia. In many of these settings, antenatal screening and treatment for these infections and preeclampsia are often unavailable or late, inconsistently or incorrectly administered, and poorly resourced.

Innovations in diagnostics might include simplified, rapid, multiplexed point-of-care tests [22], especially those that definitively identify and differentiate bacterial and viral sepsis [23–25], or routine use of biomarkers in low-resource settings to identify which women might experience premature labor [26] or predict who might suffer from preeclampsia/eclampsia [27,28]. Similarly, screening tests such as genome sequencing [29] may allow for more rapid and accurate identification of high-risk disease conditions [30–32]. In settings where there are significant physical barriers to accessing continuing care, telemonitoring may provide a potential solution. Telemonitoring is already being explored in high-income countries [33], and biometrics may be used to monitor ongoing care for sick infants and mothers. Tools designed to facilitate remote screening and monitoring will include noninvasive devices [34] for detection of conditions such as hypoxia in children [35] or hypertension in pregnant women [36]. If such tools can be designed to be both affordable and easily administered by minimally trained health workers, they may prove pivotal in encouraging prompt care seeking and thus reduce the burden of preventable or easily treatable illnesses.

Other technological innovations with the potential to have a major impact on mortality and morbidity relate to the use of safe blood products [37–39]. The supply of safe blood products is often a rate-limiting step to survival in many low- and middle-income countries, and supply often falls far short of demand. Work is currently underway on synthetic blood substitutes [40,41] that could be stored at room temperature for extended periods and may be universally compatible with all blood types [42,43]. Such products could thus be safely administered in emergency situations and in communities where supply, screening, typing, and storage of blood are a challenge.

## Commodities and Supply Chain

Innovative technologies to improve the quality, longevity, and usability of pharmaceuticals and the vaccine cold chain will likely play a major role in improving survival.

For example, the World Health Organization recommends oxytocin as the drug of choice for addressing postpartum hemorrhage, the leading cause of maternal death in low- and middle-income countries [44,45]. However, given the heat-labile nature of oxytocin, there are ongoing concerns about the stability and quality of oxytocin in tropical or subtropical climates in countries, where there may be challenges to keeping oxytocin in the cold chain consistently during transport and storage. Oxytocin is an injectable drug, and in-country policies restricting administering injections to certain categories of providers serve as an additional barrier to its use. There is work underway to produce heat-stable formulations of oxytocin, as well as research exploring alternative delivery mechanisms for oxytocin, such as the sublingual and inhaled route. This circumvents the current need where only providers trained in injection techniques can administer this. In addition, to help providers assess the potency of oxytocin at the point of use, the UN Commission on Life-Saving Commodities (UNCoLSC) is supporting efforts to pilot the inclusion of time-temperature indicators (TTIs) on vials of oxytocin [46]. The TTIs, similar to vaccine vial monitors, change color under cumulative exposure to heat and light, indicating potential issues with potency, as well as providing cues for which vials of oxytocin should be utilized first [47].

Another drug, magnesium sulphate (MgSO_4_) has been identified by the World Health Organization as the most effective, low-cost anticonvulsant for the treatment of severe preeclampsia or eclampsia [48], one of the major causes of maternal morbidity and mortality globally, yet this intervention remains widely underused [49,50]. In part, intravenous or intramuscular administration and a complex series of calculations and dosing have been key barriers to widespread use. To address this, PATH has identified a range of promising technology solutions that are currently being tested, including the use of a dilution bottle, dosing and dilution mobile app use, simplified regimens including ready-to-use MgSO_4_ packs separately containing 20% and 50% drug concentrations, and a reusable, electricity-free, low-cost infusion delivery system. Additionally, one new drug delivery platform, via rectally administered gel, is being explored and trialed [51,52].

## Addressing Demand-Side Barriers to Care Seeking and Care Provision

Demand-side barriers refer to those barriers that affect women and constrain their ability to seek care. These include, among others, poverty, poor health status, illiteracy, language, customs, lack of information regarding the availability of health services and providers, and limited control over household resources [53]. There are many innovations attempting to address demand-side issues, including the use of community health workers, community-based volunteers, peer support groups, and even electronic health (e-health: the use of information technology and communications within the health system, including laptops, netbooks, personal digital assistants [PDAs], mobile cell phones, or patient monitors), and mobile health (m-health: used mainly to describe how a healthl professional is supported by a mobile device such as a phone or PDA, which provides treatment and public health information—e.g., using short message service [SMS] or wireless technology) technologies [54,55]. These latter two are likely to play a prominent role in the post-2015 RMNCH agenda. According to Agarwal and Labrique, “MHealth strategies may have the potential to improve neonatal (and maternal) survival by catalyzing and improving the delivery of interventions of known efficacy, improving access to information and modifying demand for quality services, and enabling the provision of targeted care, where and when these benefits are needed the most” [56]. For example, Rwanda was able to demonstrate a 27% increase in facility deliveries after the introduction of SMS text messages to community health workers in the case of emergencies [57]. In Kenya, the use of mobile phones to monitor and document birth weight within 7 days of delivery significantly increased timely infant weight monitoring [58]. M-health strategies also include such things as smart phone-based treatment algorithms for remote health workers [59], cell phone-based chronic disease management [60], and the use of health management information systems (HMIS) to provide feedback for quality improvement. The potential for m-health strategies to change the face of MNCH is enormous; however, few m-health initiatives have moved beyond the pilot stage. Challenges include an insufficient evidence base for scale-up, issues of compatibility with existing systems, absence of standards for context-specific adoption, and a lack of ownership [61]. Similarly, Piette et al. conclude that “preliminary evidence shows that e-health systems can have a beneficial impact on the process of clinical care in low- and middle-income countries. However, more studies, particularly to examine the key information needs of health-care workers as well as the effects of e-health services on patient outcomes, are required in resource-poor settings” [62].

## Improving Supply-Side Issues, Especially for Marginalized Populations

Supply-side issues in RMNCH typically refer to such things as the availability of trained, culturally sensitive providers in well-supplied facilities that are physically accessible to women seeking care. Poor quality provision, maltreatment, inadequate referral systems for emergency obstetric care, lack of transportation, and the disconnect between communities and facilities often serve as barriers to care seeking [53,63]. One aspect of supply-side innovation involves bringing care to women where they are, rather than expecting women to obtain all of their care in a facility. While this is challenging and rather inefficient for complex issues requiring specialized care, it is an important area of exploration for some of the less complex issues in MNCH.

Sri Lanka more than halved its maternal and neonatal mortality by ensuring access to midwives, particularly for the rural communities [64]. Ghana developed “CHPS compounds” (community-based health planning and services programs) where an auxiliary nurse trained in basic delivery care is available in a community-based compound. Results suggest that such a model has increased the percent of women obtaining skilled delivery [65]. Many other countries have introduced similar user-centric measures, such as Ethiopia with the Health Development Army [66] and India with accredited social health activists [67].

One supply-side issue that is essential for addressing future global health needs is identifying mechanisms that simplify the process of administering medications to patients in need. The recent publication of effective simplified antibiotic regimens for infants aged 2 months and less with presumed sepsis and pneumonia [68] is stimulating enquiry into alternative dosing, route, and duration that antibiotics can be given in the treatment of presumed newborn sepsis. Such changes may allow for outpatient oral administration—or single intramuscular injections—of medications that could once only be administered via continuous intravenous (IV) administration, for example.

## Financing

Innovations in the financing of health care currently occur at several altitudes. Macrolevel innovations include such things as the Vaccine Independence Initiative (VII), which was launched in 1991 to assist low- and middle-income countries by decoupling procurement of vaccines from their payment. The VII effectively allows countries to use UNICEF as a purchasing agent, creating higher volume orders and minimizing the impact of individual nations’ poor credit [69]. The Global Fund’s Debt2Health initiative allows nations to swap existing debts for grants and convert loans to grants when certain performance targets are met [70]. While initially used mostly in HIV, tuberculosis, and malaria programs, such a financing mechanism has enormous potential for addressing deficits in MNCH as governments seek debt forgiveness.

Perhaps the most commonly discussed “innovation” in financing is the need for universal health care (UHC), primarily to reduce or eliminate user fees for pregnant and postpartum mothers. UHC is increasingly being regarded as an overarching goal for health in the post-2015 development agenda [71], yet the challenges in defining, funding, and implementing UHC require creative solutions that may vary by country. For example, the BRICS nations of Brazil, Russia, India, China, and South Africa have all signed on to the idea of UHC, but implementation has proven extremely variable and country specific [72]. Nonetheless, in countries where UHC or other programs have reduced or eliminated user fees, service utilization has increased significantly, although not always in a “pro-poor” manner and not always in a way that has translated to measurable improved outcomes among mothers and their babies [73–75].

Smaller-scale innovations in financing have included examples such as vouchers for pregnant women to visit health facilities, conditional cash transfers for mothers who engage in target behaviors [76], and even performance-based incentives for traditional birth attendants who bring their clients to facilities for antenatal, delivery, and postnatal care [77]. All of these efforts have demonstrated improvements in antenatal care attendance, facility delivery, vaccinations, and, in some cases, incidence of low birth weight [76]. One issue that such schemes typically lack is a plan for sustainability. Who will pay for the vouchers when the bilateral donor shifts priorities or when ODA falters? Thus, an important innovation for the next 20 years will be to develop models for sustaining the financial incentives that appear to be effective in getting women to seek reproductive health care.

One interesting financial innovation is the Global Financing Facility (GFF), a new mechanism with a goal to be the principal financing mechanism to accelerate and end preventable MNCH mortality by 2030. Although still in its formative phase, preliminary themes for its utilization include financing that is (1) scaled up, with 3–5 year investments supported by broader and longer term investment strategies; (2) smart, leveraging value for money; (3) sustainable, with a focus on helping countries seek innovative ways of mobilizing resources; and (4) accountable, such that every pregnancy, every birth, and every death is registered and counted. The GFF is a partnership formed initially by the governments of Norway, Canada, and the US and housed within the World Bank [12].

## Better Monitoring and Measurement, Linked to Accountability and Real-Time Feedback for Quality Assurance

Measurement is critical to the success and failure of any RMNCH endeavor. Without it, we are unable to assess the impact of programmatic efforts, care provision, or interventions, nor can we be assured that we are working toward desired targets. While there have been improvements over the past decade in global data collection and availability, there remains a paucity of robust MNCH data [78,79]. For example, it is estimated that as of 2007, only 30% of the global population lived in countries with complete vital registration systems [80]. By 2012, 57 million children (or 40% of all births) remained nonregistered by their first birthday [81], and another 15% of births occurred in countries with no vital registration data at all. Virtually all data being examined to inform national- and global-level policy change are retrospective, and virtually all are being examined 3–7 years after collection. Thus, the global community is effectively driving while looking through the rear-view mirror, rather than having real-time feedback and accurate projections to ensure more effective and efficient forward motion. Testing of real-time data collection with immediate feedback is underway, and modalities to optimally synthesize, analyze, and test these modalities to strengthen system-level, real-time data utilization are critical to progress in global health.

## Challenges to Maximum Uptake and Effectiveness of Innovations

There are many challenges to maximum uptake and effectiveness of the RMNCH innovations described here. A one-size-fits-all approach to introduction and scale-up is unlikely to be successful, given the heterogeneous nature of the innovations themselves. However, innovations will only reach their potential if they take a deliberate user-centric focus that includes an understanding of care-seeking behaviors and social and cultural preferences of women and communities, the skill, capabilities, and resources available to health care providers, and the current national and local policies regarding medical procedures that can be conducted by these providers [63,82–85]. A second challenge relates to how innovations are integrated with existing programs and within the continuum of care and beyond. Integrating approaches across the lifespan, across platforms for intervention (e.g., reinforcing the same messages at schools, churches, community centers, and health centers), and across the community-to-facility continuum will be critical for eventual success. A final challenge relates to the environment in which innovations are introduced. Only interventions which occur in an enabling environment and address the current social, medical, and logistic context can be effectively implemented and successfully scaled up for maximum public health impact [86–88].

Many of the challenges delineated above are magnified during humanitarian situations. The Ebola outbreak strained already fragile health care systems, further complicating access and delivery of routine services [89]. Even formerly robust health care systems are strained by conflict, with reversal of earlier successes; for example, the polio outbreak in Syria in 2013 triggered an outbreak response in a region that had been free of the disease for 15 years [90]. Yet, it is in precisely such environments that innovations are most crucially needed and where, counter to the challenges described above, they may be most readily adopted. Innovations which reduce complexity, streamline service delivery, minimize human resource workload, and offset infrastructural demands [91] can fundamentally alter the impact of humanitarian situations on mothers and their children.

### Conclusion

Over the next 20 years, additional emerging areas in which these transformative innovations will likely have a deep impact will include completing the unfinished MDG agenda [92], tackling stillbirth, adolescent health and preconception care, mental health, ensuring exclusive breast milk feeding, promoting integrated early childhood development practices from birth, and focusing on care involving populations such as the urban poor and displaced peoples in emergency settings.

Innovations matter. Innovations have powerful potential to result in transformative change when technologies are coupled with system-level, condition-sensitive enabling environments that support advocacy and political will, sufficient investment, a large and well-trained workforce, opportunity for real-time feedback, and quality improvement.

Given the trajectory and lessons learned from the MDGs, there is reason for cautious optimism for the future of RMNCH.

## Abbreviations

BCG: Bacillus Calmette–Guérin
BRICS: Brazil, Russia, India, China, and South Africa
CHPS: community-based health planning and services
DAC: Development Assistance Committee
DPT: diphtheria-tetanus-pertussis
e-health: electronic health
EPI: expanded program on immunizations
GAVI: Global Alliance for the Vaccine Initiative
GFF: Global Financing Facility
GNI: gross national income
HMIS: health management information systems
IV: intravenous
MDG: Millennium Development Goal
m-health: mobile health
MNCH: maternal, newborn, and child health
ODA: official development assistance
PDA: personal digital assistant
RMNCH: reproductive, maternal, newborn, and child health
SDG: Sustainable Development Goal
SMS: short message service
TTI: time-temperature indicator
UHC: universal health care
UNCoLSC: United Nations Commission on Life-Saving Commodities
VII: Vaccine Independence Initiative
